# Cytocompatibility and bioactive potential of AH Plus Bioceramic Sealer: An *in vitro* study

**DOI:** 10.1111/iej.13805

**Published:** 2022-08-11

**Authors:** José Luis Sanz, Sergio López‐García, Francisco Javier Rodríguez‐Lozano, María Melo, Adrián Lozano, Carmen Llena, Leopoldo Forner

**Affiliations:** ^1^ Department d'Estomatologia, Facultat de Medicina I Odontologia Universitat de València Valencia Spain; ^2^ Department of Dermatology, Stomatology, Radiology and Physical Medicine, Faculty of Medicine, Morales Meseguer Hospital University of Murcia Murcia Spain

**Keywords:** AH Plus Bioceramic Sealer, AH Plus sealer, bioactivity, biocompatibility, Endosequence BC sealer

## Abstract

**Aim:**

To assess the cytocompatibility and bioactive potential of the new calcium silicate cement‐based sealer AH Plus Bioceramic Sealer (AHPbcs) on human periodontal ligament stem cells (hPDLSCs) compared with the epoxy resin‐based sealer AH Plus (AHP) and the calcium silicate cement‐based sealer Endosequence BC Sealer (ESbcs).

**Methodology:**

Standardized sample discs and 1:1, 1:2 and 1:4 eluates of the tested materials were prepared. The following assays were performed: surface element distribution via SEM–EDX, cell attachment and morphology via SEM, cell viability via a MTT assay, cell migration/proliferation via a wound‐healing assay, osteo/cemento/odontogenic marker expression via RT‐qPCR and cell mineralized nodule formation via Alizarin Red S staining. HPDLSCs were isolated from extracted third molars. Comparisons were made with hPDLSCs cultured in unconditioned (negative control) or osteogenic (positive control) culture media. Statistical significance was established at *p* < .05.

**Results:**

A higher peak of Ca^2^+ was detected from ESbcs compared with AHPbcs and AHP in SEM–EDX. Both AHPbcs and ESbcs showed significantly positive results in the cytocompatibility assays (cell viability, migration/proliferation, attachment and morphology) compared with a negative control group, whilst AHP showed significant negative results. Both AHPbcs and ESbcs exhibited an upregulation of at least one osteo/odonto/cementogenic marker compared with the negative and positive control groups. Both ESbcs and AHPbcs showed a significantly higher calcified nodule formation than the negative and positive control groups, indicative of their biomineralization potential and were also significantly higher than AHP group.

**Conclusion:**

AH Plus Bioceramic Sealer exhibited a significantly higher cytocompatibility and bioactive potential than AH Plus and a similar cytocompatibility to that of Endosequence BC Sealer. Endosequence BC Sealer exhibited a significantly higher mineralization potential than the other tested sealers. The results from this *in vitro* study act as supporting evidence for the use of AH Plus Bioceramic Sealer in root canal treatment.

## INTRODUCTION

Root canal treatment involves the chemical–mechanical disinfection of the root canal system and its subsequent filling to ensure an adequate seal (Li et al., 2014). The materials placed inside the root canal should be dimensionally stable, biocompatible and present adequate handling properties (Donnermeyer et al., 2019). The most commonly used materials for such purpose are gutta‐percha and root canal sealers (Kishen et al., 2016; Vishwanath & Rao, 2019).

A wide variety of root canal sealer compositions are available, such as zinc oxide‐eugenol, epoxy resin and calcium silicate cement sealers (Sfeir et al., 2021). These materials differ in terms of their setting reactions, which take place by chelate formation, polymer formation by addition and hydration, respectively (Komabayashi et al., 2020). The physicochemical and biological properties of these sealers also differ (Silva et al., 2019, 2021).

Endodontic sealers are placed inside the root canal and may extrude to a variable extent during root canal treatment through the apical and/or secondary foramina into the surrounding supporting tissues (Aminoshariae & Kulild, 2020). Therefore, sealers should exhibit an adequate biocompatibility, i.e. they should not induce an adverse reaction or response from biological tissues upon contact (Ferreira et al., 2021). The same applies on a cellular level, wherein surrounding cellular populations should not experience a decrease in their viability, migration/proliferation or differentiation (da Silva et al., 2017). In other words, root canal sealers should exhibit an adequate cytocompatibility and absence of cytotoxicity.

Periodontal ligament stem cells (PDLSCs), a subgroup of dental stem cells (DSCs) with a mesenchymal phenotype (Bartold & Gronthos, 2017), are in contact with root canal sealers. These cells possess a multilineage differentiation potential and may play a crucial role in the healing process of existing periapical lesions (Gay et al., 2007; Seo et al., 2004). Consequently, the extrusion of a root canal sealer with adequate biological properties should not hinder these cells.

Previous evidence has demonstrated that resin‐based or resin‐containing sealers and cements often are cytotoxic toward various cell subpopulations (Collado‐González, Tomás‐Catalá, et al., 2017; Manaspon et al., 2021). The well‐known AH Plus sealer (Dentsply DeTrey GmbH) has shown a negative effect on periodontal ligament stem cell viability, migration/proliferation and attachment in various *in vitro* studies (Oh et al., 2020; Rodríguez‐Lozano et al., 2019) compared with a negative control group.

Conversely, calcium silicate cement‐based sealers have adequate biocompatibility and bioactive properties when cultured together with PDLSCs (Rodríguez‐Lozano et al., 2017; Zheng et al., 2020). These sealers have shown the ability to induce the precipitation of a layer of hydroxyapatite on their surface (Kim et al., 2015). which may form a mineral attachment to dentin tissue (Vallittu et al., 2018). The same term is used to describe their positive influence on cell plasticity, e.g. by favouring the osteo/odonto/cementogenic differentiation of PDLSCs which, in turn, may result in an enhanced repair process and resolution of periapical lesions (Sanz, Guerrero‐Gironés, et al., 2021).

AH Plus Bioceramic Sealer (Maruchi) was introduced into the market as a pre‐mixed tricalcium silicate cement‐based sealer. According to its distributor (Dentsply Sirona USA), this new sealer presents a faster setting time, lower solubility, lower film thickness and higher radiopacity than the Endosequence BC Sealer (Brasseler). However, to the authors' knowledge, the biological properties of the new AH Plus Bioceramic Sealer towards PDLSCs have not been elucidated.

Accordingly, the aim of the present *in vitro* study is to assess the cytocompatibility and bioactive properties of AH Plus Bioceramic Sealer on hPDLSCs compared with the classic AH Plus and Endosequence BC Sealer.

## MATERIALS AND METHODS

The manuscript of this *in vitro* study has been written in accordance with the Preferred Reporting Items for Laboratory studies in Endodontology (PRILE) 2021’ guidelines (Nagendrababu et al., 2021a).

### Preparation of material extraction mediums

Sample discs were prepared for each of the tested materials (*n* = 30 in total): AH Plus Bioceramic Sealer (AHPbcs), AH Plus (AHP) and Endosequence BC Sealer (ESbcs). The number of discs was based on the protocol from a previous study with similar methodology (Sanz, López‐García, et al., 2021). Material data (composition, manufacturer and batch number) are listed in Table 1. Materials were placed into 5‐mm diameter and 2‐mm high sterile (ultraviolet radiation, 15 min) cylindrical rubber molds with Hank's balanced salt solution (HBSS; H6648; Sigma Aldrich) and set in an incubator at 37°C, 5% CO_2_ and 95% humidity for 48 hours. AHP is presented in a two‐component paste/paste format. Accordingly, pastes A and B were mixed following its manufacturer's instructions. Both AHPbcs and ESbcs are presented in an injectable syringe (pre‐mixed) format and were directly placed into the rubber moulds.

**TABLE 1:** Data on the tested materials

Material	Manufacturer	Composition^a^	Batch number
AH Plus Bioceramic Sealer	Manufactured by Maruchi Distributed by Dentsply DeTrey GmbH	Zirconium dioxide (50%–75%), tricalcium silicate (5%–15%), dimethyl sulfoxide (10%–30%), lithium carbonate (<0.5%), thickening agent (<6%)	KS210728
AH Plus	Dentsply DeTrey GmbH	Paste A: bisphenol‐A epoxy resin, bisphenol‐F expoxy resin, calcium tungstate, zirconium oxide, silica, iron oxide pigments Paste B: dibenzyldiamine, aminoadamantane, tricyclodecane‐diamine, calcium tungstate, zirconium oxide, silica, silicone oil	2105000678
Endosequence BC Sealer	Manufactured by Innovative Bioceramix Distributed by Brasseler	Zirconium dioxide (35%–45%), tricalcium silicate (20%–35%), dicalcium silicate (7%–15%), calcium hydroxide (1%–4%)	21001SP

^a^ The concentration of each component of the tested materials is presented as a percentage by weight (WT%) within brackets. Data were extracted from the respective Material Safety Data Sheets, if available.

To simulate clinical conditions, where cells are in contact with the silicate cement‐based sealers, extracts or eluates were obtained from each of the materials, following the International Standard ISO 10993‐5 guidelines (ISO, 2009). The eluates of the different materials were extracted in sterile conditions, using Dulbecco Modified Eagle's Medium (DMEM) (Gibco) with 10% of foetal bovine serum (FBS) as an extraction vehicle. The extraction procedure was as follows: the tested materials were immersed in the culture medium for 24 h at 37°C in a humid atmosphere containing 5% CO_2_. In accordance with the ISO standard, the ratio between the surface of the sample and the volume of the medium was 1.5 cm^2^/ml. The extraction medium was collected at the end of this period and filtered through a 0.22‐μm syringe filter (Merck Millipore). Thereafter, in order to study the effect of the concentration of each material, various dilutions (1:1, 1:2 and 1:4 v/v) of these extraction media were prepared using fresh complete DMEM medium (Rodríguez‐Lozano et al., 2019).

### Material surface element distribution: SEM‐EDS analysis

The previously prepared material sample discs were selected for the analysis (*n* = 5 per material). After the incubation period, the set material discs were coated with carbon under a CC7650 SEM Carbon Coater Unit (Quorum Technologies Ltd.). The superficial element distribution of the coated discs was then individually examined in a scanning electron microscopy (SEM) unit (Jeol 6100 EDAX; Jeol Inc.) attached to an energy dispersive spectroscopy (EDS) system (INCA 350 EDS; Oxford Instruments) for the elemental analysis.

### Isolation, culture and characterization of human PDLSCs

The human PDLSC (hPDLSC) extraction protocol had been approved by the Human Research Ethics Committee from *Universidad de Murcia* (ID: 2199/2018), following the Helsinki Declaration guidelines. HPDLSCs were isolated from healthy third molars from 18–30‐year‐old patients (*n* = 10), which had been extracted for orthodontic or periodontal reasons; with written informed consent. The molar sample size was selected in accordance with a previous study with similar methodology (Sanz, López‐García, et al., 2021).

Extracted molars were immediately placed in Minimum Essential Medium with Alpha modifications (α‐MEM; Gibco, Invitrogen) supplemented with 1% penicillin/streptomycin (Sigma Aldrich) and amphotericin B (Fungizone; Sigma Aldrich) and stored at 4°C. The teeth were rinsed thrice with phosphate‐buffered saline (PBS) (Gibco), and the periodontal tissues were scraped from the surface of the middle and apical thirds of their roots. Periodontal tissues were sliced into smaller fragments and digested with Collagenase type I solution (3 mg/ml; Gibco) for 1 h at 37°C. The periodontal cells were seeded in α‐MEM supplemented with 10% foetal bovine serum (FBS; Sigma Aldrich) and 1% penicillin/streptomycin (Sigma Aldrich).

Before their use in the *in vitro* experimentation, hPDLSC characterization was performed following the International Society of Cellular Therapy (ISCT) guidelines (Dominici et al., 2006), to confirm their mesenchymal nature. The process was as follows: cells were analysed under flow cytometry (FACSCalibur Flow Cytometry System; BD Biosciences), and the high expression of the mesenchymal stem cell (MSC)‐specific surface markers CD73, CD90 and CD105 and low expression of the hematopoietic markers CD34, CD45, CD14 and CD20 were confirmed. This was performed in accordance with similar studies in the field (Collado‐González, García‐Bernal, et al., 2017; Oh et al., 2020). Additionally, the resultant characterized hPDLSCs were cultured in different media (osteogenic/adipogenic/chondrogenic) (Miltenyi Biotec) to confirm their trilineage mesenchymal differentiation. Both the mesenchymal nature and trilineage differentiation potential of the cells used were confirmed by a previous study performed by the present research group (Rodríguez‐Lozano et al., 2019). For the subsequent *in vitro* experimentation, cells from passages 2–4 were used, as performed in previous similar studies (Sanz, López‐García, et al., 2021).

### Material cytotoxicity: MTT assay

Material cytotoxicity was assessed for the different eluates (1:1, 1:2 and 1:4) of AHP, AHPbcs and ESbcs cultured with hPDLSCs (test groups) and compared with hPDLSCs cultured in unconditioned growth medium (negative control group). This analysis was performed via a 3‐(4,5‐dimethylthiazol‐2‐yl)‐2,5‐diphenyltetrazolium bromide (MTT) assay, as previously reported by similar studies (Rathinam et al., 2021). In brief, hPDLSCs were seeded onto 96‐well plates with 180 μl of DMEM and stored for 24 h at 37°C, 5% CO_2_ and 95% humidity. The material eluates were placed in the culture medium with 1 × 10^4^ hPDLSCs (*n* = 3). An MTT reagent (Sigma Aldrich) was added for 4 h, following its manufacturer's instructions. When a purple precipitate was detectable, Dimethylsulfoxide (DMSO) (Sigma‐Aldrich) was added to each well (100 μl/well), and plates were covered and kept in dark conditions for 4 h to solubilize the formazan crystals produced by viable cells, after reducing the MTT reagent. After 24, 48 and 72 h of culture, light absorbance per well was recorded by means of a microplate reader (ELx800; Bio‐Tek Instruments) at 570 nm wavelength. Culture media with fresh eluates from the respective groups were replaced every 3 days.

### Cell migration/proliferation: Horizontal wound healing assay

HDPLSC migration/proliferation was assessed after culture in growth medium with the eluates (1:1, 1:2 and 1:4) of AHP, AHPbcs and ESbcs and compared with the cells cultured in unconditioned growth medium (negative control group) via a wound healing assay. HPDLSCs were seeded onto 6‐well plates (2 × 10^5^ cells per well; *n* = 3 for each experimental condition) and left to proliferate until cell confluency was reached. Then, a superficial scratch wound was made on each cell monolayer using a 200‐μl sterilized pipette tip, and each well was rinsed thrice to remove any remaining cell debris. Wound closure/healing was assessed for all experimental conditions in triplicate (test groups and negative control) at 24, 48 and 72 h. At each time‐point, the percentage of open wound area was quantified for each of the samples by means Image J software (National Institutes of Health). Migration rates were presented as percentage areas of relative wound closure (RWC) to account for width variations amongst the scratch wounds. RWC values were calculated as follows: RWC = (wound closure area [in pixels]/total number of pixels) × 100. Results are expressed as the percentage of the total wound area thrice relative to the total wound area at 0 h for each respective well.

### Cell morphology and attachment: SEM visualization

Sealer discs were made using the previously described methods (*n* = 5 for each sealer). The surface of the discs was seeded with 5 × 10^4^ hDPSCs and cultured in normal growth medium for 72 h. Cells were fixed with 4% glutaraldehyde (Sigma‐Aldrich) in PBS for 4 h. The cells were dehydrated using a series of gradually increasing ethanol dilutions (30 to 90% v/v) and treated with hexamethyldisilazane (Sigma‐Aldrich) for 5 min. Finally, cells were air‐dried, sputter‐coated with gold and palladium and examined using a SEM (Jeol 6100 EDAX; Jeol Inc.) at 100×, 300× and 1500× magnifications.

### Cell osteo/cemento/odontogenic gene expression: RT‐qPCR assay

The osteo/cemento/odontogenic marker expression of hPDLSCs cultured together with the materials was assessed via real‐time quantitative polymerase chain reaction (RT‐qPCR), as a measurement of cell differentiation. Twenty‐thousand hPDLSCs per well were seeded onto 12‐well plates (*n* = 3) and incubated for 3, 7, 14 and 21 days with undiluted (1:1) sealer‐conditioned medium from the two calcium silicate‐cements (test groups: AHPbcs or ESbcs), in unconditioned culture medium (negative control groups) or in osteogenic differentiation medium (positive control; OsteoDiff media; Miltenyi Biotec). Culture media with fresh eluates from the respective groups were replaced every 3 days. The undiluted sealer‐conditioned medium was prepared by immersing the previously conditioned standardized sealer discs in culture medium (DMEM; Gibco) for 24 h. AHP was excluded from the marker expression assay because of its negative results in the hPDLSC viability, migration/proliferation and attachment assays. Total RNA was extracted from hPDLSCs using the RNeasy Mini Kit (Qiagen). One μg of RNA was reverse transcribed for first‐strand complementary DNA (cDNA) synthesis via iScript™ Reverse Transcription Supermix for RT‐qPCR (Bio‐Rad Laboratories Inc.). Both processes were performed following their respective manufacturers' kit instructions.

The primer sequences for the differentiation markers used for the assay were as follows (5′–3′): Cementum attachment protein or CAP (forward: TTTTTCTGGTCGCGTGGACT, reverse: TCACCAGCAACTCCAACAGG), cementum protein 1 or CEMP1 (forward: GGGCACATCAAGCACTGACAG, reverse: CCCTTAGGAAGTGGCTGTCCAG), alkaline phosphatase or ALP (forward: TCAGAAGCTCAACACCAACG, reverse: TTGTACGTCTTGGAGAGGGC), runt‐related transcription factor 2 or RUNX2 (forward: TCCACACCATTAGGGACCATC, reverse: TGCTAATGCTTCGTGTTTCCA), bone sialoprotein or BSP (forward: TGCCTTGAGCCTGCTTCCT, reverse: CTGAGCAAAATTAAAGCAGTCTTCA), amelogenin X or AMELX (forward: CACCCTGCAGCCTCATCACC, reverse: GTGTTGGATTGGAGTCATGG).

Differentiation marker expression was measured relative to the expression of the housekeeping gene glyceraldehyde 3‐phosphate dehydrogenase (GAPDH), with the following sequence (5′‐3′): (forward: TCAGCAATGCCTCCTGCAC, reverse: TCTGGGTGGCAGTGATGG). To calculate the relative gene expression, the standardized 2−ΔΔCT method was used (Livak & Schmittgen, 2001).

### Cell mineralization/calcified nodule formation: Alizarin Red S Staining

An Alizarin Red S Staining (ARS) assay was performed to assess hPDLSC calcified nodule formation in contact with the tested sealers (AHPbcs, AHP and ESbcs), as a measurement of their biomineralization potential. Twenty‐thousand hPDLSCs per well were seeded onto 12‐well plates (*n* = 3) and left to proliferate until confluency was reached. The cells were then transferred into undiluted (1:1) sealer‐conditioned medium and cultured for 21 days. After the culture period, the samples were rinsed with foetal bovine serum and fixed with 70% ethanol for 1 h. Then, samples were stained with 2% Alizarin Red solution (Sigma Aldrich) for 30 min in controlled conditions (dark ambient and room temperature) and solubilized using 10% cetylpyridinium chloride monohydrate solution (Sigma‐Aldrich). Lastly, absorbance values of the samples were measured using Synergy H1 multi‐mode microplate reader (BioTek) at 570 nm. For this assay, both a negative control (hDPSCs cultured in unconditioned growth medium [DMEM; Gibco]) and a positive control (hDPSCs cultured in osteogenic medium (OsteoDiff; Miltenyi Biotec) were used for reference.

### Statistical analysis

All the experimental conditions and measurements were performed in triplicate for each of the tested sealers (AHPbcs, AHP and ESbcs). Data are expressed as mean ± standard deviations (SD). The normality in the distribution of the data was previously confirmed via a Q‐Q plot. Data were analysed using one‐way anova and Tukey's *post hoc* test using Graph‐Pad Prism v8.1.0 (GraphPad Software). To perform the one‐way anova test, we grouped the data by time (24 h, 48 h and 72 h) and analysed them independently. Each dilution was considered an independent treatment. Statistical significance was considered at *p* < .05.

## RESULTS

Data (mean and standard deviations) from the biological assays are presented in the Supplementary Material, as follows: Table S1 (MTT assay), Table S2 (wound healing assay), Table S3 (Alizarin Red S staining) and Table S4 (RT‐qPCR).

### SEM‐EDS analysis

**FIGURE 1:**
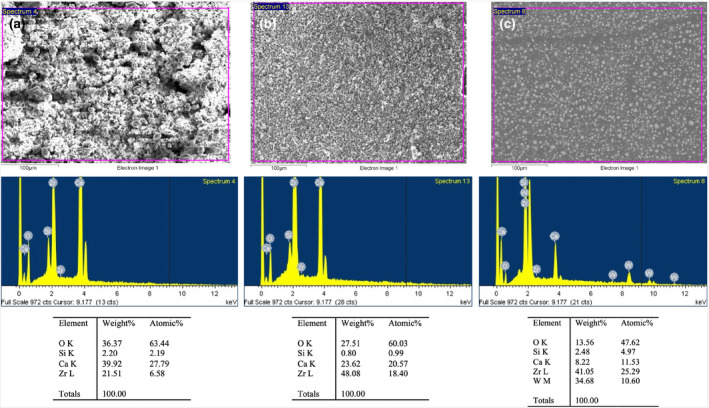
Results from the SEM‐EDS analysis for the tested sealers (ESbcs [column a], AHPbcs [column b], AHP [column c]). The first row illustrates SEM images of each material (scale bar: 100 μm). The second row shows the EDS elemental spectra. The third row lists the elements present per sealer by weight and atomic weight.

SEM‐EDS analysis revealed the superficial element distributions of the root canal sealers (ESbcs, AHPbcs and AHP; Figure 1). ESbcs and AHPbcs displayed a superficial crystalline structure, whilst particles on the smoother AHP surface were spherical. The elements O, Si, Ca and Zr were detected in all samples. Interestingly, a higher peak of Ca^2+^ was detected in ESbcs compared with AHPbcs and AHP, whereas a higher peak of zirconium (Zr) was observed in AHPbcs compared with the other material samples. Tungsten (W) was detected only in the AHP samples.

### MTT assay

**FIGURE 2:**
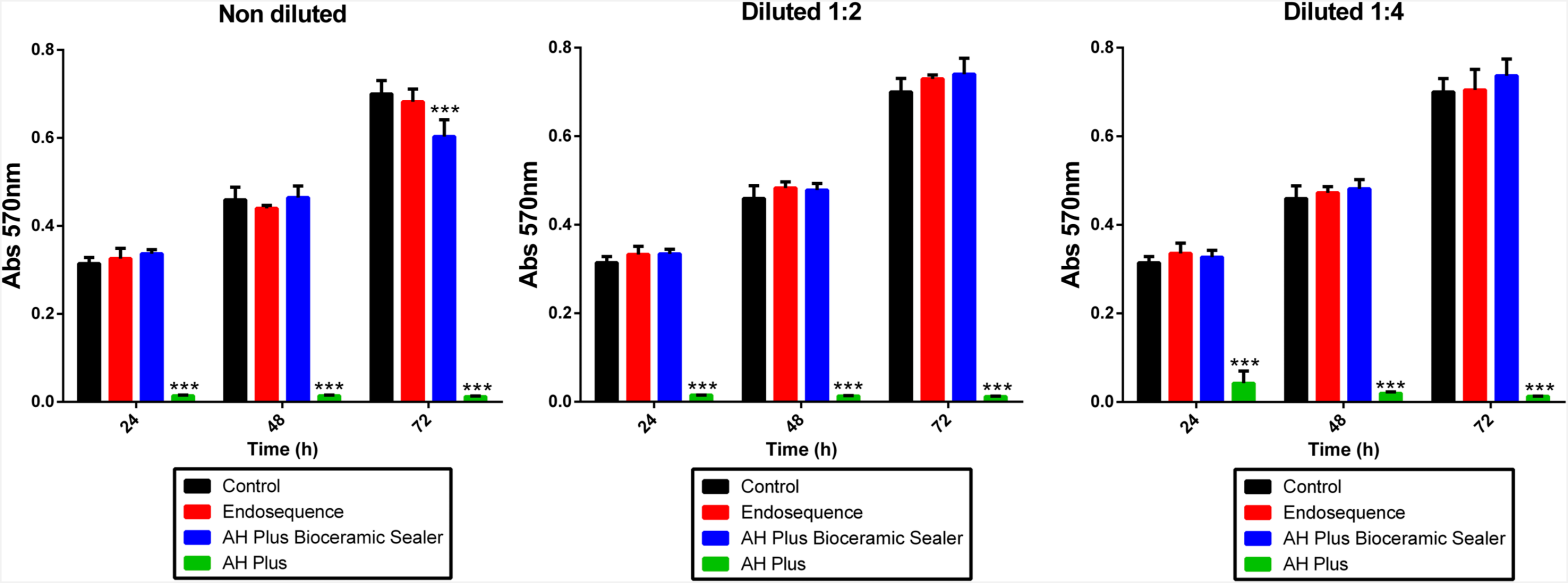
Results from MTT assay for the different eluates (1:1, 1:2, 1:4) of the tested sealers (ESbcs, AHPbcs and AHP) after 24, 48 and 72 h of culture with hPDLSCs. Data are presented absorbance values (570 nm) at the different measurement time‐points, compared with the negative control group. ***p < .001 (One‐way anova analysis).

The MTT assay revealed an adequate cell viability from all eluates of ESbcs and AHPbcs at all the tested time points (24, 48 and 72 h of culture), similar to that of the control group; the 1:1 AHPbcs‐treated cells, however, exhibited a significantly lower viability than the control group (*p* < .001). AHP‐treated cells exhibited a significantly lower viability compared with the control group after 24, 48 and 72 h of culture (*p* < .001; Figure 2).

### Wound healing assay

**FIGURE 3:**
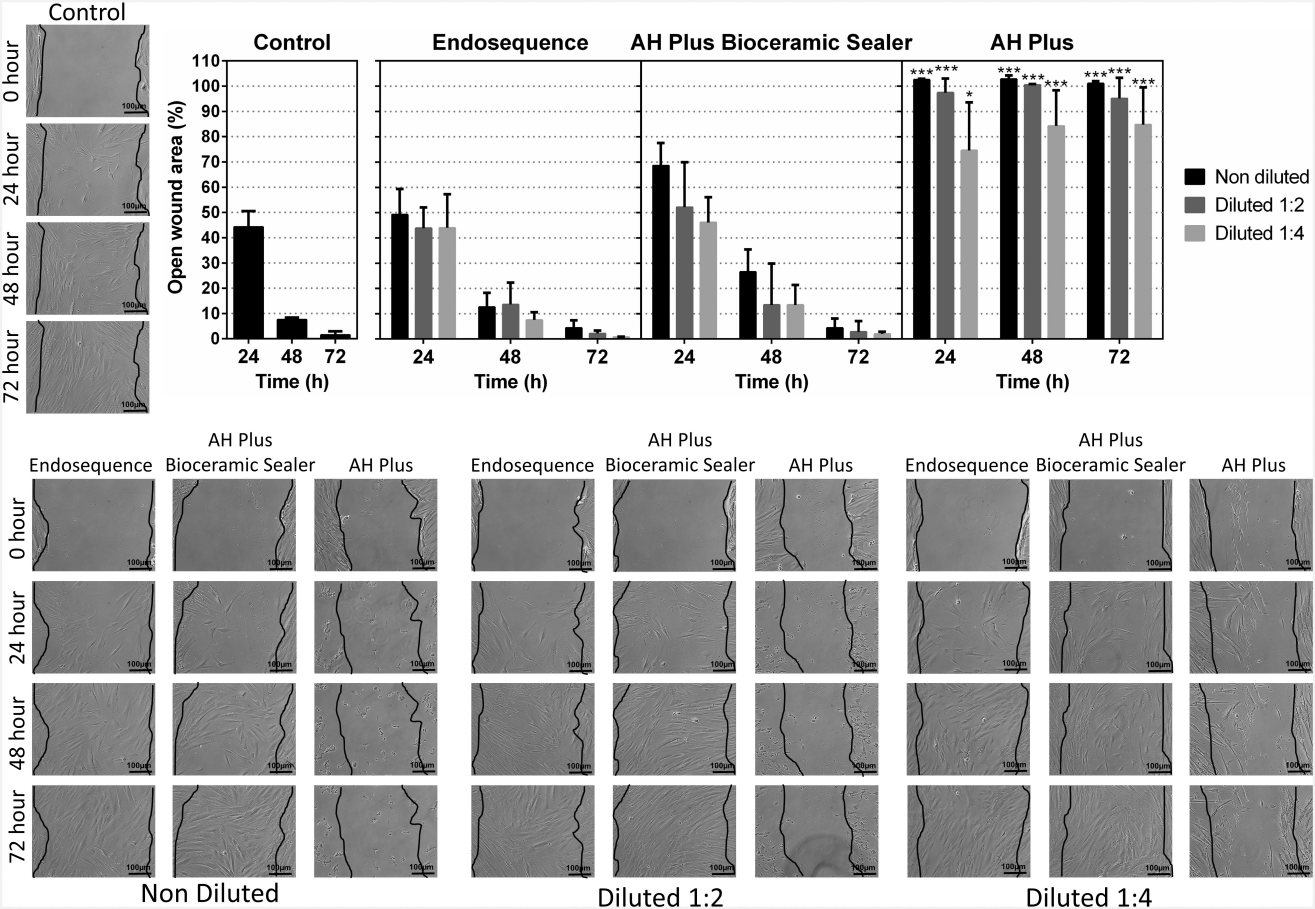
Results from the wound healing assay for the different eluates (1:1, 1:2, 1:4) of the tested sealers (ESbcs, AHPbcs and AHP) after 24, 48 and 72 h of culture with hPDLSCs. Graphical results are presented as percentages of open wound areas at the different measurement time points, compared with the negative control group. ***p < .001 (One‐way anova analysis). Images: Scale bar 100 μm.

Human periodontal ligament stem cells cultured with all the eluates of AHPbcs and ESbcs exhibited similar migration to that of the control group at all time‐points (24, 48 and 72 h) in the wound healing assay. Similar to the cytotoxicity assay, cells cultured with AHP exhibited a significantly lower migration compared with the control group after 24, 48 and 72 h of culture (*p* < .001; Figure 3).

### SEM visualization for hPDLSCs

**FIGURE 4:**
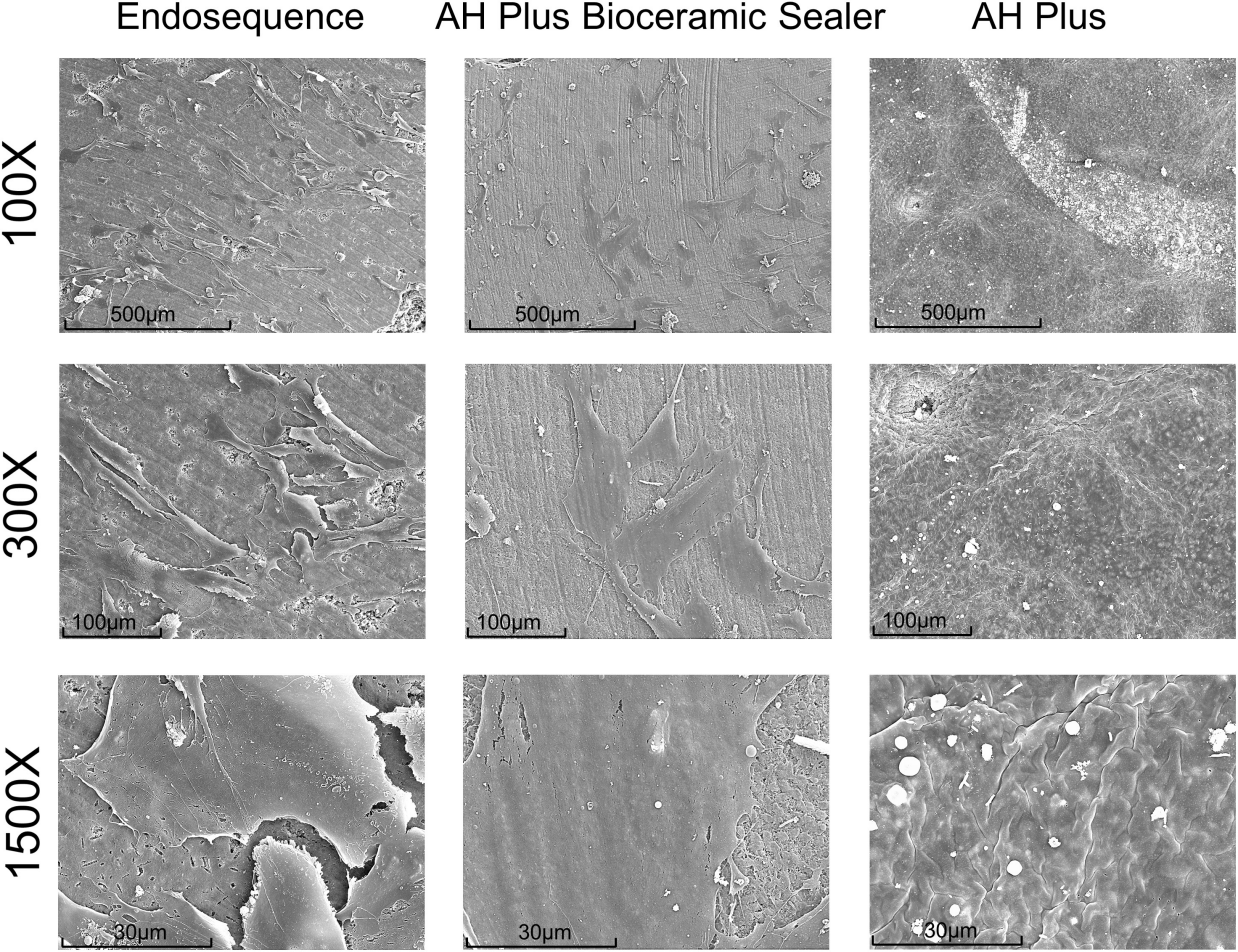
Results from the SEM visualization after 72 h of culture of hPDLSCs seeded onto the surface of the tested material sample discs (ESbcs, AHPbcs and AHP). Magnifications: 100×, 300× and 1500×. Scale bars: 500 μm, 100 μm and 30 μm.

SEM images revealed differing hPDLSC morphologies and attachment on the surface of the root canal sealer discs (ESbcs, AHPbcs and AHP). HPDLSCs seeded onto the surface of ESbcs and AHPbcs sample discs exhibited a spindle‐like elongated morphology, intense growth and spread. It should be highlighted that a higher number of attached cells were visible on the surface of ESbcs compared with that of AHPbcs. Conversely, the surface of AHP sample discs had a low quantity of cells and debris, indicating cell death (Figure 4).

### RT‐qPCR assay

**FIGURE 5:**
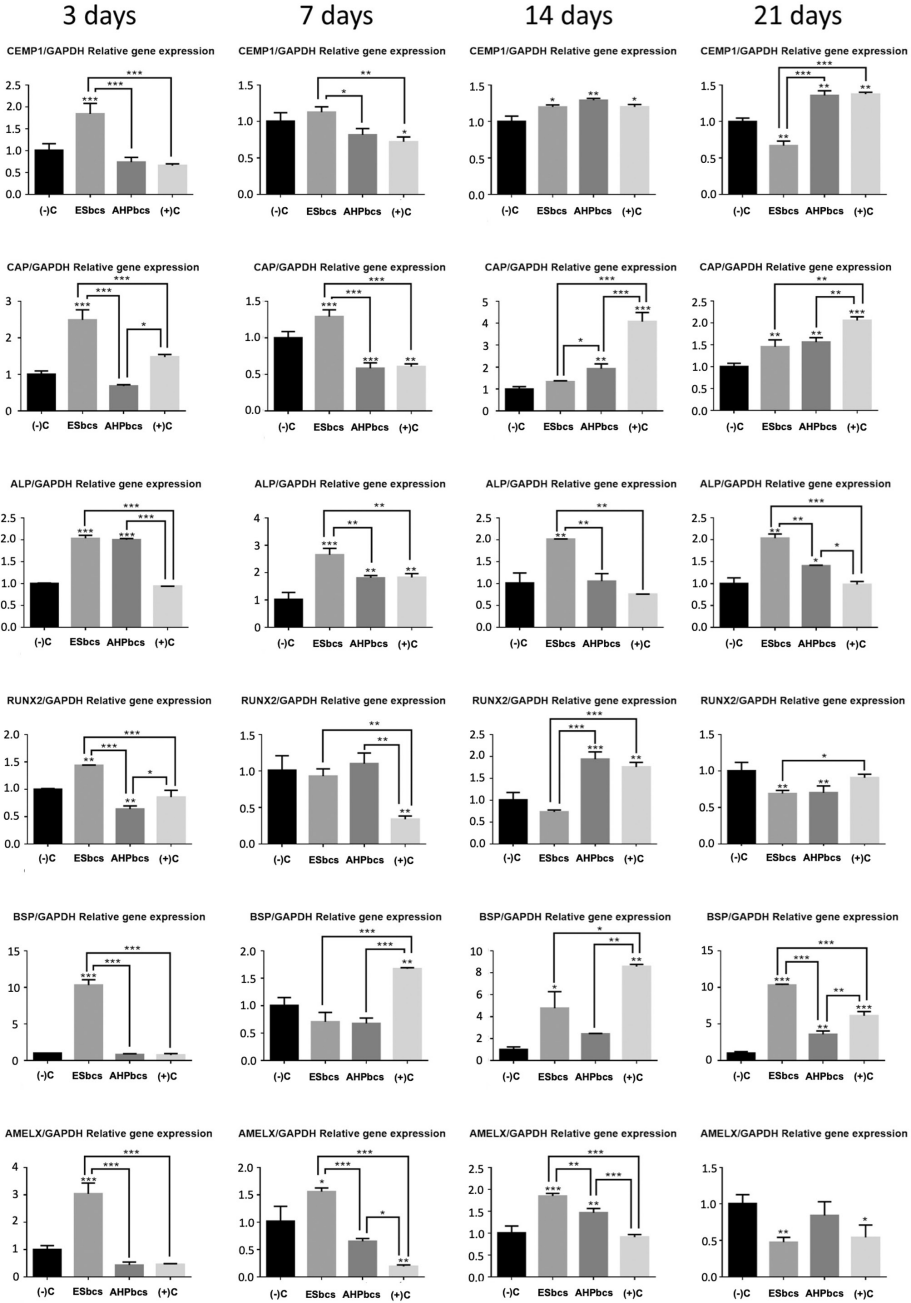
Results from the analysis of hPDLSCs osteo/odonto/cementogenic marker expression via RT‐qPCR after 3, 7, 14 and 21 days of culture with DMEM (negative control), ESbcs, AHPbcs, or Osteodiff (postive control). *p < .05; **p < .01; ***p < .001 (Two‐way anova analysis). Asterisks above the bars indicate a significant difference with the negative control group. Asterisks above the lines indicate a significant difference between the groups which the line is connecting.

The RT‐qPCR assay for the assessment of osteo/odonto/cementogenic marker expression from hPDLSCs cultured with the tested materials (ESbcs or AHPbcs) produced a wide variety of results (Figure 5).

AHPbcs‐treated cells exhibited a significantly higher early expression (3 and 7 days of culture) of ALP compared with the negative control group and a significantly higher late expression (14 and 21 days of culture) of CEMP1, CAP, ALP (*p* < .01 at 21 days), RUNX2 and BSP. Compared with the positive control group, AHPbcs‐treated cells exhibited a significantly higher early expression of ALP and RUNX2 and a late expression of ALP and AMELX.

ESbcs‐treated cells exhibited a significantly higher early expression of all the tested markers compared with the negative and positive control groups, and a significantly higher late expression of CEMP1, CAP, ALP, BSP and AMELX compared with the negative control group. ESbcs‐treated cells also exhibited a significantly higher late expression of ALP and BSP compared with the positive control group.

When comparing the two calcium silicate cement sealers, ESbcs‐treated cells exhibited a significantly higher early expression of all the tested markers and a significantly higher late expression of ALP, BSP and AMELX. On the other hand, AHPbcs‐treated cells exhibited a significantly higher late expression of CEMP, CAP and RUNX2.

### Alizarin Red S Staining

**FIGURE 6:**
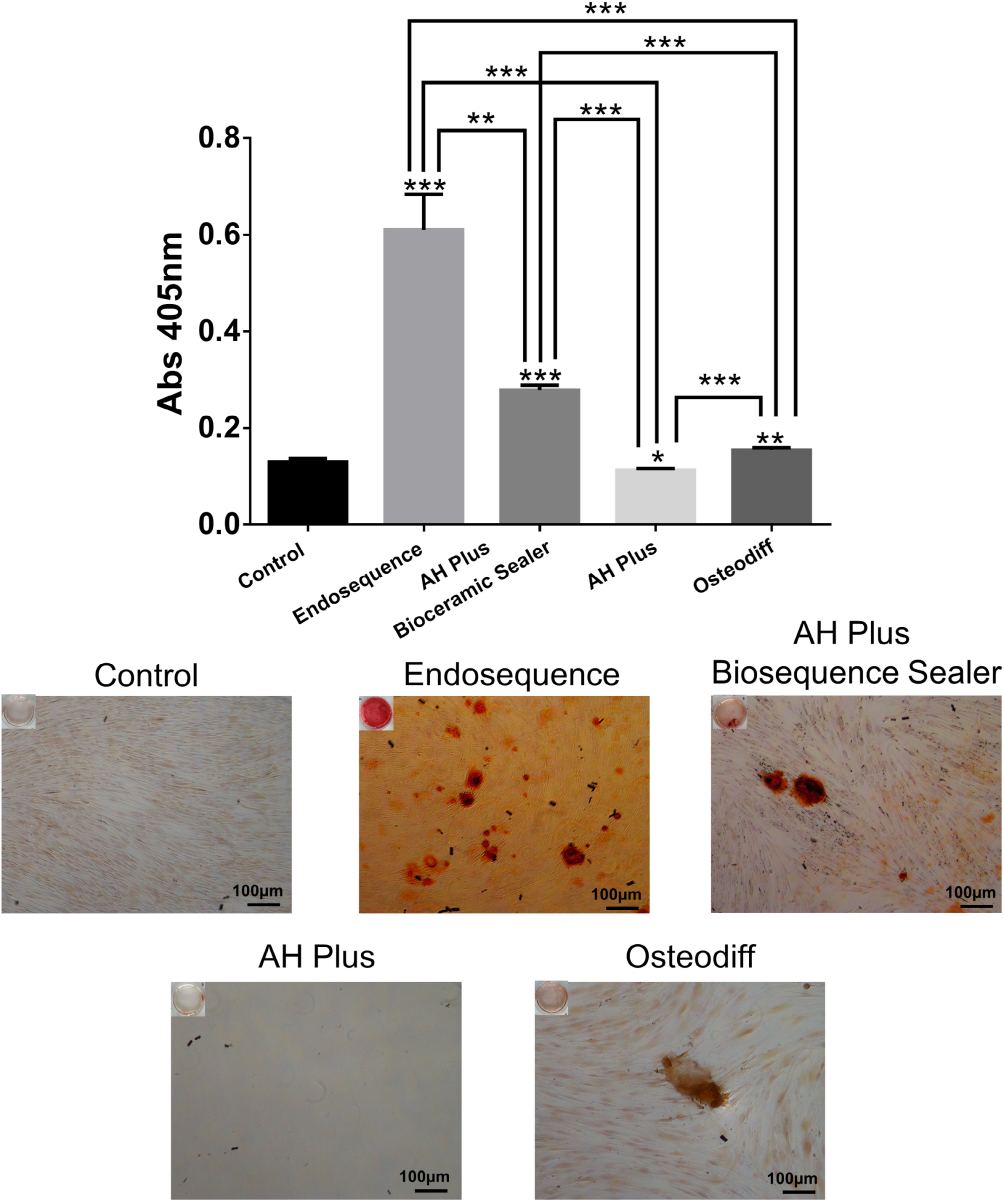
Results from the Alizarin Red S staining of hPDLSCs after 21 days of culture with DMEM (negative control), ESbcs, AHPbcs, AHP or Osteodiff (positive control). *p < .05; **p < .01; ***p < .001 (Two‐way anova analysis). Asterisks above the bars indicate a significant difference with the negative control group. Asterisks above the lines indicate a significant difference between the groups which the line is connecting.

Results from the cell mineralization assay are presented in Figure 6. Both AHPbcs and ESbcs‐treated hPDLSCs exhibited a significantly higher mineralized nodule formation than the negative and positive control groups (*p* < .001). The AHP‐treated hPDLSCs exhibited a significantly lower mineralization compared with the negative and positive control groups (*p* < .05 and *p* < .001; respectively). As expected, cells cultured in osteogenic medium (Osteodiff; positive control) showed a significantly higher mineralization (*p* < .01) than those cultured in unconditioned medium (negative control). ESbcs‐treated cells showed a significantly higher calcified nodule formation than those treated with AHPbcs (*p* < .01).

## DISCUSSION

New biomaterial formulations are constantly being introduced into the market for clinical use in the field of endodontics (Camilleri et al., 2022). Currently, calcium silicate cement‐based materials are increasing in use amongst clinicians (Careddu et al., 2021). The recently introduced AH Plus Bioceramic Sealer is presented as a potential alternative to the classic AH Plus and other calcium silicate cement‐based sealers such as Endosequence BC Sealer. Accordingly, the aim of the present study was to assess its cytocompatibility and bioactive properties on hPDLSCs and compare them with the aforementioned sealers.

**FIGURE 7:**
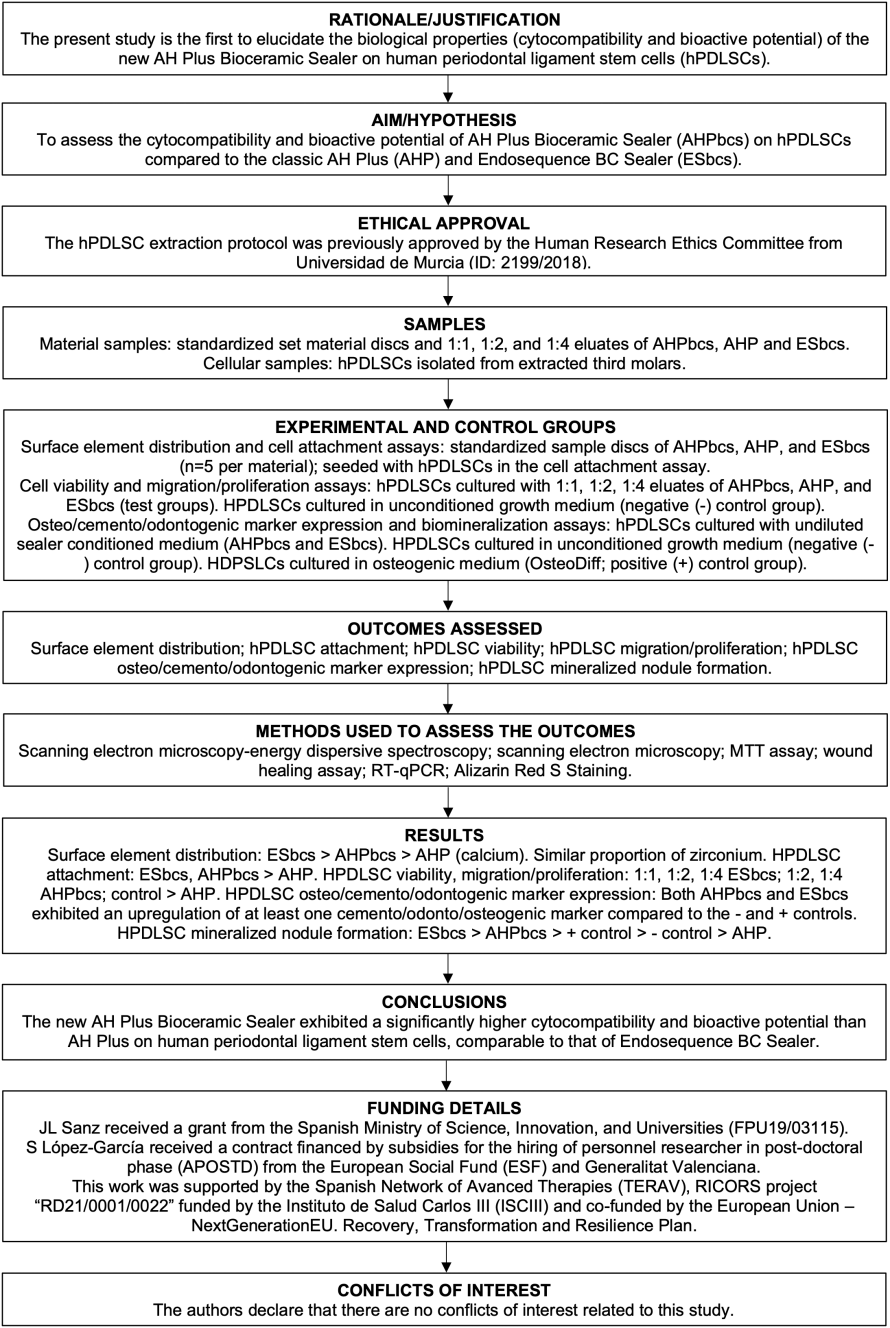
Preferred Reporting Items for Laboratory studies in Endodontology (PRILE 2021)‐based flowchart.

*In vitro* study designs like the present one offer a consistent analysis of the main biological properties of dental materials cultured together with cellular subpopulations with which they will come into contact during their clinical use and thus may predict their clinical behaviour (Pedano et al., 2020). However, as a limitation of the present work, several variables could affect the differences between the results observed under laboratory and clinical conditions, such as variations in pH, variations in oxygen levels or the patients' immune response (Sanz, Guerrero‐Gironés, et al., 2021). Nevertheless, as a strength of the present work, the use of standardized material sample preparation and biological assay procedures (ISO 10993‐5, 2009) results in an increased reproducibility of the study design and consequently an increased homogeneity between studies. Lastly, following specific reporting guidelines enhances the comparability between studies with similar methodologies. In the present study, the recently introduced PRILE guidelines were followed for such purpose (Nagendrababu et al., 2021b). Accordingly, the main steps of this work have been depicted in the PRILE 2021 flowchart (Figure 7).

Biocompatibility and cytocompatibility assays are useful to confirm the positive or negative response of biological tissues and cellular populations. In the present study three cytocompatibility assays were performed: MTT assay, as a measure of cell viability; wound healing assay, as a measure of cell proliferation/migration; and SEM visualization, as a measure of cell morphology and attachment. All the cytocompatibility assays produced concordant results. Both calcium silicate cement‐based sealers (ESbcs and AHPbcs) exhibited an adequate cytocompatibility compared with a negative control group. These results are in accordance with previous studies on the biological properties of calcium silicate cement‐based sealers as a group of dental materials (Mann et al., 2022; Park et al., 2021; Zordan‐Bronzel et al., 2019). The main components tricalcium silicate and a radiopacifying agent (zirconium oxides) have been shown to be biocompatible in previous studies (Campi et al., 2022).

The epoxy resin‐based sealer AHP showed signs of cytotoxicity on hPDLSCs, evidenced by their significant decrease in cell viability and proliferation and aberrant morphology and attachment. These results are in accordance with previous studies on several cell subpopulations (Saygili et al., 2017; Rodríguez‐Lozano et al., 2019). The resinous component in endodontic sealers or in resin‐modified calcium silicate cements has been associated with the increased cytotoxicity (Bakir et al., 2022; Sanz, Soler‐Doria, et al., 2021; Silva et al., 2013).

SEM is useful for the evaluation of the superficial morphology and texture of materials. Regarding calcium silicate cement‐based materials, it can also be suitable for the evaluation of their hydration (Anthrayose et al., 2021). Both ESbcs and AHPbcs displayed a crystalline surfaces, unlike the epoxy resin sealer. AHPbcs exhibited a denser and more homogeneous microstructure. The hydration extent can influence the properties of cements (Camilleri, 2007) and thus may account for the differences observed in the mineralization assay.

Energy dispersive spectroscopy was used to examine the elements present on the surface of the samples. However, EDS only exhibits the distribution of elements on the sample's surface. Other complementary techniques such as X‐ray diffraction (XRD) analysis or attenuated total reflection–Fourier transform infrared (ATR‐FTIR) spectroscopy are needed to identify calcium hydroxide peaks and other crystalline phases in hydraulic cements after setting (Ferreira et al., 2021).

Differences in the elemental composition of the tested biomaterials may account for their differing biological properties. For example, both calcium silicate cement‐based sealers disclosed tricalcium silicate in their composition, but ESbcs also incorporates dicalcium silicate. Additionally, tricalcium silicate only represents 5%–15% in weight in AHPbcs, whilst ESbcs contain 20%–35% of tricalcium silicate and 7%–15% of dicalcium silicate. Differences in the composition of calcium silicate cement‐based materials affect their behaviour (Watson et al., 2014). This explains the significantly higher mineralization exhibited by ESbcs‐treated cells compared with those treated with AHPbcs in the ARS assay.

As expected, both ESbcs and AHPbcs contained more calcium and oxygen than AHP. Calcium and hydroxyl ion release after hydration has been associated with the favourable biological properties of calcium silicate cement‐based endodontic biomaterials (Khalil et al., 2016). The observed Ca^2+^ peak in AH Plus can be explained by the inclusion of calcium tungstate (CaWO_4_) as a radiopacifier in its composition. The Zr observed in AHPbcs compared with ESbcs is supported by the differences in their percentage by weight (WT%) listed in their respective Material Safety Data Sheets (50%–70% vs. 35%–45%). The higher proportion of radiopacifier (ZrO_2_) in the AHPbcs correlated with differences in in the biological properties of endodontic sealers, as others have noted (Queiroz, Torres, Rodrigues, Viola, Bosso‐Martelo, Chavez‐Andrade, et al., 2021b). Thus, in future research, it could be interesting to study the biological properties of new biomaterial compositions from the perspective of the differences in radiopacifying agents and concentrations, as performed by a recent study (Queiroz, Torres, Rodrigues, Viola, Bosso‐Martelo, Chavez‐Andrade, et al., 2021a).

It should be highlighted that the inability to assess the influence of fillers, thickening agents, additives and/or vehicles can act as a limitation of the analysis of the biological properties of the tested materials from the perspective of the differences in their composition. The presence and proportion of these components in the composition of the tested materials are often regarded as confidential business information. For example, 1%–4% of calcium hydroxide is included as a non‐confidential additive in Endosequence BC Sealer. This contributed to the observed Ca^2+^ peak in the SEM‐EDS analysis. Thus, other confidential additives could explain other differences in the materials' biological properties.

Results from the RT‐qPCR assay are varied but follow a general pattern. In brief, hPDLSCS cultured with both ESbcs or AHPbcs exhibited a significant upregulation of at least one cementogenic, osteogenic and odontogenic marker compared with the negative and positive control group. These markers were assessed, based on similar studies in the field (Rodríguez‐Lozano et al., 2020; Zheng et al., 2020): CEMP1, CAP, ALP, RUNX2, BSP, and AMELX.

Cementogenic markers such as CEMP and CAP are important indicators of hPDLSC activity since they play a crucial role in the regeneration and repair of the periodontum. Specifically, an overexpression of CAP is seen during cell recruitment and differentiation during the formation of cementum tissue, whilst CEMP‐1 is involved in the regulation of the differentiation of periodontal cells (Arzate et al., 2015; Pitaru et al., 1995). Thus, the overexpression of CEMP and CAP exhibited by ESbcs and AHPbcs‐treated hPDLSCs may indicate their positive influence in cell plasticity and enhancement of the healing process of periodontal defects from lesions of endodontic origin.

BSP is a mineralized tissue‐specific marker that is highly expressed during the initial formation of bone tissue and is synthesized by osteoblasts and osteoclastic‐like cells in culture (Garcia et al., 2003; Ogata, 2008). ALP promotes bone formation by degrading inorganic pyrophosphate and generating inorganic phosphate, a crucial molecule in differentiation and mineralization of osteoblasts (Osathanon et al., 2009; Seltzer et al., 1962). Therefore, the overexpression of these markers exhibited by ESbcs and AHPbcs‐treated hPDLSCs is a complementary indicator of the positive influence of these materials in cell plasticity and differentiation into an osteoblast‐like lineage. This may reflect their potential enhancement of the process of bone tissue repair or regeneration.

RUNX2 has been reported to be essential for the later stages of tooth formation, since it is involved in the development of mineralized dental tissue (Camilleri & McDonald, 2006). Additionally, it has been reported that RUNX2 is essential for osteoblast differentiation (Bruderer et al., 2014), and that its overexpression enhances the osteogenic activity of bone marrow stromal cells (Zhao et al., 2005). AMELX encodes for amelogenin, a structural modeling protein involved in the biomineralization process of amelogenesis (Green et al., 2019). Amelogenesis results in the formation and growth of hydroxyapatite crystals (Guo et al., 2015). Thus, the upregulation of these markers adds to the evidence on the enhancement of these materials of the process of mineralized tissue formation.

An Alizarin Red S staining assay was performed as a complementary measure of the influence of the tested materials on hPDLSC mineralized tissue formation. The significantly higher calcified nodule formation exhibited by ESbcs and AHPbcs‐treated cells, compared with the negative and positive controls, provided further support to their biomineralization ability. Similar results have been obtained in previous studies on other calcium silicate cement‐based endodontic sealers (Rodríguez‐Lozano et al., 2019; Sanz, López‐García, et al., 2021). Contrarily, AHP‐treated cells showed negative results on this assay, as observed in the aforementioned studies.

Altogether, the results from the cytocompatibility and bioactivity assays point towards the positive influence of ESbcs and AHPbcs on hPDLSC viability, migration, morphology, attachment, differentiation and biomineralization; and the negative influence of AHP on the same parameters. To the authors' knowledge, this is the first study to elucidate the biological properties of AHPbcs in controlled laboratory conditions. Further studies of interest in testing the material's behaviour in animal models or clinical trials.

## CONCLUSION

The new calcium silicate cement‐based sealer AH Plus Bioceramic Sealer exhibited a significantly higher cytocompatibility and bioactive potential than the epoxy resin‐based sealer AH Plus on human periodontal ligament stem cells. The cytocompatibility of AH Plus Bioceramic Sealer was comparable with that of the calcium silicate cement‐based sealer Endosequence BC Sealer. Both calcium silicate‐based sealers exhibited a significantly higher bioactive potential compared with a negative control group. However, Endosequence BC Sealer exhibited a significantly higher mineralization potential than AH Plus Bioceramic Sealer and AH Plus. The results from this *in vitro* study act as supporting evidence for the use of AH Plus Bioceramic Sealer in root canal treatment.

## AUTHOR CONTRIBUTIONS

Investigation and methodology: Sergio López‐García, Francisco Javier Rodríguez Lozano, José Luis Sanz; supervision, visualization, conceptualization and data curation: María Melo, Leopoldo Forner, Carmen Llena; investigation, methodology and writing—original draft: José Luis Sanz, Francisco Javier Rodríguez‐Lozano; conceptualization, formal analysis, project administration, supervision, validation and writing—review and editing: Leopoldo Forner, Carmen Llena, María Melo; investigation, methodology, project administration, resources, writing—original draft, and writing—review and editing: José Luis Sanz, Francisco Javier Rodríguez‐Lozano, Leopoldo Forner. All authors have read and agreed to the published version of the manuscript.

## FUNDING INFORMATION

JL Sanz received a grant from the Spanish Ministry of Science, Innovation, and Universities (FPU19/03115). S López‐García received a contract financed by subsidies for the hiring of personnel researcher in post‐doctoral phase (APOSTD) from the European Social Fund (ESF) and Generalitat Valenciana. This work was supported by the Spanish Network of Avanced Therapies (TERAV), RICORS project ‘RD21/0001/0022’ funded by the Instituto de Salud Carlos III (ISCIII) and co‐funded by the European Union – NextGenerationEU. Recovery, Transformation and Resilience Plan.

## CONFILCT OF INTEREST

The authors declare no conflicts of interest related to this study.

## ETHICS STATEMENT

The cell extraction protocol was approved by the Human Research Ethics Committee from the University of Murcia (reference: 2199/2018).

## Supporting information


Tables S1–S4

## Data Availability

The data that support the findings of this study are available from the corresponding author upon reasonable request.
