# Supplementary material for: Cytocompatibility and bioactive potential of AH Plus Bioceramic Sealer: An in vitro study
Source: Int Endod J. 2022 Aug 11;55(10):1066–80. doi: 10.1111/iej.13805 (PMC9541143; doi:10.1111/iej.13805)
Supplement: Supplementary file 1 — Tables S1–S4 [file IEJ-55-1066-s001.docx]

Supplementary Table 1: MTT assay (Abs 570nm)

|  | 24h | | 48h | | 72h | |
| --- | --- | --- | --- | --- | --- | --- |
|  | Mean | SD | Mean | SD | Mean | SD |
| Control | 0,314 | 0,014 | 0,459 | 0,029 | 0,700 | 0,030 |
| Endosequence 1:1 | 0,326 | 0,023 | 0,440 | 0,007 | 0,682 | 0,029 |
| Endosequence 1:2 | 0,333 | 0,018 | 0,483 | 0,014 | 0,752 | 0,031 |
| Endosequence 1:4 | 0,335 | 0,023 | 0,473 | 0,014 | 0,705 | 0,047 |
| AH Plus Bioceramic Sealer 1:1 | 0,337 | 0,009 | 0,464 | 0,026 | 0,603 | 0,038 |
| AH Plus Bioceramic Sealer 1:2 | 0,343 | 0,016 | 0,479 | 0,015 | 0,747 | 0,036 |
| AH Plus Bioceramic Sealer 1:4 | 0,327 | 0,016 | 0,481 | 0,021 | 0,737 | 0,038 |
| AH Plus 1:1 | 0,014 | 0,001 | 0,014 | 0,001 | 0,012 | 0,001 |
| AH Plus 1:2 | 0,015 | 0,000 | 0,013 | 0,001 | 0,012 | 0,001 |
| AH Plus 1:4 | 0,042 | 0,027 | 0,020 | 0,004 | 0,013 | 0,001 |

Supplementary Table 2: Wound healing assay (Open wound area (%))

|  | 24h | | 48h | | 72h | |
| --- | --- | --- | --- | --- | --- | --- |
|  | Mean | SD | Mean | SD | Mean | SD |
| Control | 44,264 | 6,232 | 7,548 | 0,796 | 1,466 | 1,476 |
| Endosequence 1:1 | 49,181 | 10,171 | 12,630 | 5,607 | 4,390 | 3,029 |
| Endosequence 1:2 | 43,870 | 8,181 | 13,689 | 8,653 | 2,184 | 1,161 |
| Endosequence 1:4 | 43,958 | 13,296 | 7,565 | 2,995 | 0,779 | 0,201 |
| AH Plus Bioceramic Sealer 1:1 | 68,575 | 8,937 | 26,561 | 8,887 | 4,383 | 3,732 |
| AH Plus Bioceramic Sealer 1:2 | 52,104 | 17,863 | 13,489 | 16,398 | 2,850 | 4,212 |
| AH Plus Bioceramic Sealer 1:4 | 46,129 | 10,002 | 13,483 | 7,930 | 2,036 | 0,873 |
| AH Plus 1:1 | 102,576 | 0,392 | 102,811 | 1,437 | 101,091 | 0,880 |
| AH Plus 1:2 | 97,438 | 5,584 | 100,522 | 0,328 | 95,199 | 8,151 |
| AH Plus 1:4 | 74,587 | 19,021 | 84,277 | 14,118 | 84,812 | 14,730 |

Supplementary Table 3: Alizarin Red S Staining (Abs 405nm)

|  | Mean | SD |
| --- | --- | --- |
| Control | 0,130 | 0,007 |
| Endosequence | 0,603 | 0,015 |
| AH Plus Bioceramic Sealer | 0,278 | 0,010 |
| AH Plus | 0,113 | 0,003 |
| Osteodiff | 0,158 | 0,001 |

Supplementary Table 4: RT-qPCR assay (GENE/GAPDH Relative gene expression)

| 3 DAYS |  | Control | Endosequence | AH Plus Bioceramic Sealer | Osteodiff |
| --- | --- | --- | --- | --- | --- |
| CEMP1 | Mean | 1,008 | 1,840 | 0,738 | 0,660 |
|  | SD | 0,149 | 0,238 | 0,108 | 0,038 |
| CAP | Mean | 1,003 | 2,492 | 0,685 | 1,476 |
|  | SD | 0,090 | 0,275 | 0,033 | 0,067 |
| ALP | Mean | 1,000 | 2,032 | 2,002 | 0,938 |
|  | SD | 0,011 | 0,069 | 0,025 | 0,001 |
| RUNX2 | Mean | 1,000 | 1,438 | 0,639 | 0,856 |
|  | SD | 0,014 | 0,006 | 0,057 | 0,127 |
| BSP | Mean | 1,000 | 10,315 | 0,817 | 0,740 |
|  | SD | 0,021 | 0,745 | 0,122 | 0,211 |
| AMELX | Mean | 1,006 | 3,039 | 0,432 | 0,456 |
|  | SD | 0,136 | 0,392 | 0,114 | 0,030 |

| 7 DAYS |  | Control | Endosequence | AH Plus Bioceramic Sealer | Osteodiff |
| --- | --- | --- | --- | --- | --- |
| CEMP1 | Mean | 1,004 | 1,127 | 0,816 | 0,722 |
|  | SD | 0,116 | 0,073 | 0,087 | 0,065 |
| CAP | Mean | 1,002 | 1,291 | 0,585 | 0,606 |
|  | SD | 0,083 | 0,093 | 0,073 | 0,039 |
| ALP | Mean | 1,022 | 2,652 | 1,808 | 1,826 |
|  | SD | 0,252 | 0,237 | 0,083 | 0,137 |
| RUNX2 | Mean | 1,012 | 0,931 | 1,102 | 0,339 |
|  | SD | 0,197 | 0,100 | 0,146 | 0,044 |
| BSP | Mean | 1,005 | 0,705 | 0,676 | 1,673 |
|  | SD | 0,144 | 0,173 | 0,100 | 0,022 |
| AMELX | Mean | 1,018 | 1,557 | 0,652 | 0,190 |
|  | SD | 0,270 | 0,070 | 0,051 | 0,029 |

| 14 DAYS |  | Control | Endosequence | AH Plus Bioceramic Sealer | Osteodiff |
| --- | --- | --- | --- | --- | --- |
| CEMP1 | Mean | 1,002 | 1,198 | 1,290 | 1,198 |
|  | SD | 0,067 | 0,029 | 0,027 | 0,035 |
| CAP | Mean | 1,004 | 1,334 | 1,929 | 4,073 |
|  | SD | 0,106 | 0,043 | 0,220 | 0,417 |
| ALP | Mean | 1,013 | 2,013 | 1,055 | 0,754 |
|  | SD | 0,229 | 0,007 | 0,171 | 0,005 |
| RUNX2 | Mean | 1,009 | 0,734 | 1,938 | 1,757 |
|  | SD | 0,167 | 0,043 | 0,167 | 0,106 |
| BSP | Mean | 1,013 | 4,770 | 2,426 | 8,571 |
|  | SD | 0,231 | 1,511 | 0,049 | 0,204 |
| AMELX | Mean | 1,008 | 1,846 | 1,465 | 0,909 |
|  | SD | 0,153 | 0,062 | 0,097 | 0,058 |

| 21 DAYS |  | Control | Endosequence | AH Plus Bioceramic Sealer | Osteodiff |
| --- | --- | --- | --- | --- | --- |
| CEMP1 | Mean | 1,001 | 0,669 | 1,357 | 1,374 |
|  | SD | 0,047 | 0,063 | 0,065 | 0,026 |
| CAP | Mean | 1,002 | 1,452 | 1,562 | 2,054 |
|  | SD | 0,076 | 0,159 | 0,100 | 0,084 |
| ALP | Mean | 1,004 | 2,036 | 1,409 | 0,980 |
|  | SD | 0,129 | 0,097 | 0,013 | 0,070 |
| RUNX2 | Mean | 1,004 | 0,692 | 0,703 | 0,908 |
|  | SD | 0,114 | 0,042 | 0,093 | 0,049 |
| BSP | Mean | 1,012 | 10,320 | 3,579 | 6,125 |
|  | SD | 0,215 | 0,128 | 0,469 | 0,574 |
| AMELX | Mean | 1,005 | 0,476 | 0,842 | 0,542 |
|  | SD | 0,123 | 0,064 | 0,189 | 0,168 |
